# Anthocyanin-Rich Fraction of Black Rice Bran Extract Protects against Amyloid β-Induced Oxidative Stress, Endoplasmic Reticulum Stress, and Neuronal Apoptosis in SK-N-SH Cells

**DOI:** 10.3390/ph17081039

**Published:** 2024-08-07

**Authors:** Sivanan Sivasinprasasn, Jiraporn Tocharus, Sugunya Mahatheeranont, Sarun Nakrat, Chainarong Tocharus

**Affiliations:** 1 Department of Anatomy, Faculty of Medicine, Chiang Mai University, Chiang Mai 50200, Thailand; dymesiva@gmail.com; 2 Office of Research Administration, Chiang Mai University, Chiang Mai 50200, Thailand; 3 Department of Physiology, Faculty of Medicine, Chiang Mai University, Chiang Mai 50200, Thailand; jtocharus@gmail.com; 4 Department of Chemistry, Faculty of Science, Chiang Mai University, Chiang Mai 50200, Thailand; sugunya.w@gmail.com (S.M.); sarun_n@cmu.ac.th (S.N.); 5 Center of Excellence for Innovation in Chemistry, Faculty of Science, Chiang Mai University, Chiang Mai 50200, Thailand

**Keywords:** Alzheimer’s disease, amyloid-beta, apoptosis, ER stress, anthocyanins, cyanidin-3-*O*-glucoside

## Abstract

Alzheimer’s disease (AD) is the most common neurodegenerative disorder in the aging population. An accumulation of amyloid plaques and neurofibrillary tangles causes degeneration of neurons, leading to neuronal cell death. The anthocyanin-rich fraction of black rice (*Oryza sativa* L. variety “Luem Pua”) bran (AFBRB), extracted using a solution of ethanol and water and fractionated using Amberlite XAD7HP column chromatography, contains a high anthocyanin content (585 mg of cyanidin-3-*O*-glucoside and 24 mg of peonidin-3-*O*-glucoside per gram of the rich extract), which has been found to reduce neurodegeneration. This study focused on the neuroprotective effects of AFBRB in Aβ_25–35_-induced toxicity in the human neuroblastoma cell line (SK-N-SH). SK-N-SH was exposed to Aβ_25–35_ (10 µM) to induce an AD cell model in vitro. Pretreatment with AFBRB (0.1, 1, or 10 µg/mL) or C3G (20 µM) was conducted for 2 h prior to the treatment with Aβ_25–35_ (10 µM) for an additional 24 h. The results indicate that AFBRB can protect against the cytotoxic effect of Aβ_25–35_ through attenuation of intracellular ROS production, downregulation of the expression of the proteins Bax, cytochrome c, cleaved caspase-9, and cleaved caspase-3, upregulation of the expression of Bcl-2 in the mitochondrial death pathway, and reduction in the expression of the three major markers of ER stress pathways in similar ways. Interestingly, we found that pretreatment with AFBRB significantly alleviated Aβ-induced oxidative stress, ER stress, and apoptosis in SK-N-SH cells. This suggests that AFBRB might be a potential therapeutic agent in preventing neurodegenerative diseases.

## 1. Introduction

Alzheimer’s disease (AD) has become the most common type of neurodegenerative disease in the elderly. AD is mainly characterized by the extracellular accumulation of beta-amyloid (Aβ) plaques, neurofibrillary tangles (NFT), and neuronal loss [1,2,3]. Previous studies have reported that Aβ plays an important role in the onset and aggravation of AD. The excessive aggregation of Aβ peptides and the deposition of amyloid plaques demonstrate neurotoxic effects which lead to neuronal cell apoptosis [4,5,6]. It has been reported that Aβ generates the production of reactive oxygen species (ROS) such as superoxide anion (O_2_^−^) and impairs mitochondrial redox activity, which leads to conditions of oxidative stress [7]. The activation of oxidative stress and the intrinsic apoptotic pathway play critical roles in mediating Aβ-induced neural cell death through the mitochondrial apoptotic pathway [8,9]. Mitochondria are crucial to the regulation of apoptotic processes. The Aβ-induced apoptotic pathway involving mitochondria has been shown to be regulated by proapoptotic members such as Bax and Bak and antiapoptotic members such as Bcl-2 and Bcl-XL [10,11,12]. Activation of mitochondrial membrane permeability, which leads to the induced translocation of proapoptotic proteins from cytosol to the mitochondrial membrane, causes the inhibition of anti-apoptotic proteins and releases cytochrome c from mitochondria to cytosol, with subsequent activation of caspase-9 and caspase-3 and hence, progression to apoptosis [13,14]. Moreover, Aβ disrupts the function of the endoplasmic reticulum (ER), resulting in the accumulation of unfolded proteins in the ER and consequently causing ER stress [15,16]. During conditions of ER stress, glucose-regulated protein-78 (Grp78), the master of the chaperone proteins, accumulates within the ER, which has released from three of the ER transmembrane sensors, thus activating the production of transcription factor 6—also known as ATF6—protein kinase R-like ER kinase (PERK), and inositol-requiring enzyme 1 (IRE1) [17,18]. Excessive chaperone proteins bind to misfolded proteins, thereby activating ATF6, PERK, and IRE, leading to apoptosis by stimulating the expression of the C/EBP homologous protein (CHOP), which is known as a Bcl-2 inhibitor [19]. Several studies show that Aβ promotes the generation of ROS, which are involved in the regulation of calcium in the ER. The disturbance of calcium homeostasis within the ER, caused by an increase in intracellular ROS, contributes to the development of ER stress. Calpain, a calcium-activated protease in the cytosol, can induce the activation of caspase-12. Subsequently, the caspase-12 activates the caspase cascade, which is related to the ER stress-induced apoptosis pathway [20,21,22,23].

There are more than 200 black rice varieties found worldwide [24]. Rice bran is a by-product of the rice milling process. Colored rice bran contains a large amount of anthocyanins responsible for color and bioactivities. Anthocyanins are usually the main ones, with about 90% being cyanidin-3-glucoside (C3G) and small traces of peonidin-3-glucoside (P3G), cyanidin-3-rutinoside (C3R), and malvidin-3-glucoside (M3G) in black rice [25]. The anthocyanin composition of rice bran varies greatly in different colors, while that of rice bran with the same color is also slightly different depending on rice varieties, growing environment, and cropping conditions. Black glutinous rice, *Oryza sativa* L. var. Indica “Luem Pua”, a traditional sticky rice from the north of Thailand, has been reported to have a high anthocyanin content. In this study, an anthocyanin-rich fraction of black rice bran (AFBRB) was extracted from Luem Pua rice. Our extract contains a large amount of C3G and other anthocyanins (Supplementary Materials). A previous study reported that anthocyanin-rich fraction of black rice bran (AFBRB) could alleviate kidney dysfunction in obese rats by attenuating oxidative stress and apoptosis [26]. This is of interest in evaluating the protective mechanisms of AFBRB. The aim of the present study was to investigate the effects of AFBRB on Aβ-induced neuronal damage in SK-N-SH cells and to explore its possible mechanism through oxidative stress, ER stress, and the apoptosis pathway.

## 2. Results

### 2.1. Effects of AFBRB on Cell Viability Induced by Aβ_25–35_ in SK-N-SH Cells

**Figure 1:**
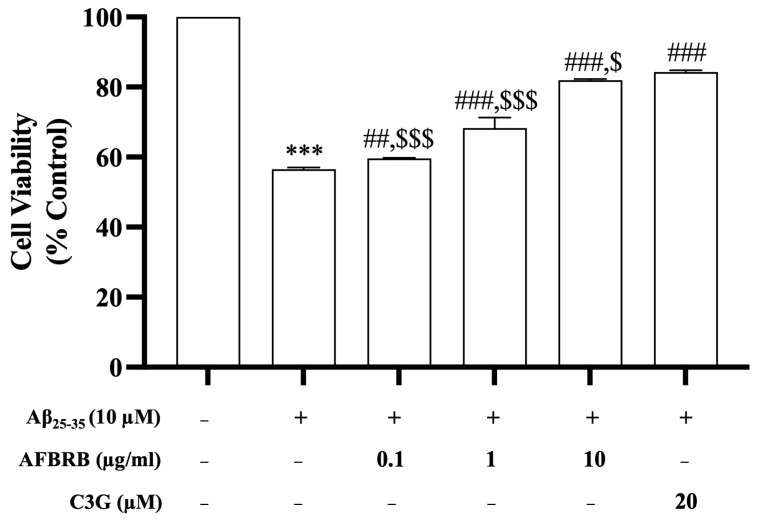
The protective effect of AFBRB and C3G against Aβ_25–35_-induced cell death in the SK-N-SH cells as determined using the MTT assay. The data are expressed as mean ± SD of six independent experiments. *** *p* < 0.001 versus control group; ^##^ *p* < 0.01, and ^###^ *p* < 0.001 versus 10 µM Aβ_25–35_-treated group; ^$^ *p* < 0.05, and ^$$$^ *p* < 0.001 versus C3G.

To investigate the effects of AFBRB or C3G against Aβ_25–35_-induced cell death, SK-N-SH cells were pretreated with 0.1, 1, or 10 µg/mL of AFBRB or 20 µM of C3G for 2 h before the addition of 10 µM of Aβ_25–35_ for 24 h. The results showed the Aβ_25–35_-induced cell death of approximately 56.52 ± 0.50% of cells when compared with the control group. Treatment with AFBRB or C3G can attenuate the cytotoxicity of Aβ_25–35_ and significantly increase cell viability in a dose-dependent manner. The percentage of cell viability upon pretreatment with AFBRB at concentrations of 0.1, 1, or 10 µg/mL or 20 µM of C3G before being treated with Aβ_25–35_ was 59.62%, 68.29%, 81.92%, and 84.32%, respectively, as shown in Figure 1.

### 2.2. Effect of AFBRB on Intracellular ROS Production Induced by Aβ_25–35_ in SK-N-SH Cells

**Figure 2:**
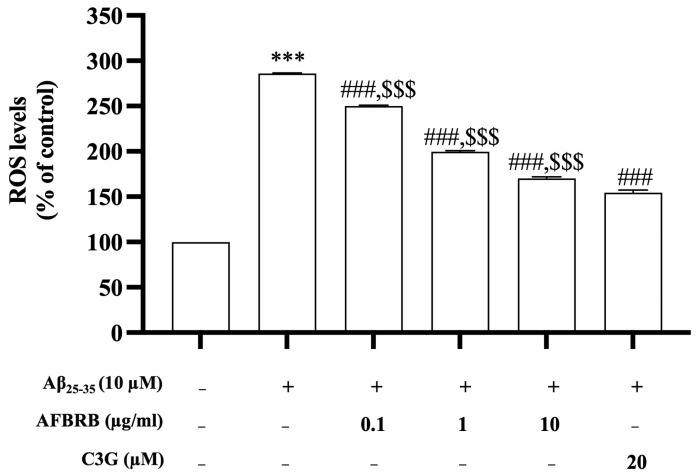
The effect of AFBRB and C3G on Aβ_25–35_-induced intracellular ROS production in the SK-N-SH cells as determined using the DCFDA assay. The data are expressed as mean ± SD of six independent experiments. *** *p* < 0.001 versus control group; ^###^ *p* < 0.001 versus 10 µM Aβ_25–35_-treated group; ^$$$^ *p* < 0.001 versus C3G.

The levels of intracellular ROS production were evaluated in Aβ_25–35_-induced SK-N-SH cells. Cells were pretreated with AFBRB or C3G for 2 h before the addition of 10 µM of Aβ_25–35_ for 24 h. Results showed that Aβ_25–35_ alone significantly increased the levels of ROS by approximately 285.90% compared with the control group. Pretreatment with AFBRB at concentrations of 0.1, 1, or 10 µg/mL or 20 µM of C3G significantly reduced the levels of production of ROS in a dose-dependent manner (Figure 2).

### 2.3. Effect of AFBRB on Aβ_25–35_-Induced Apoptosis via the Mitochondrial Death Pathway in SK-N-SH Cells

**Figure 3:**
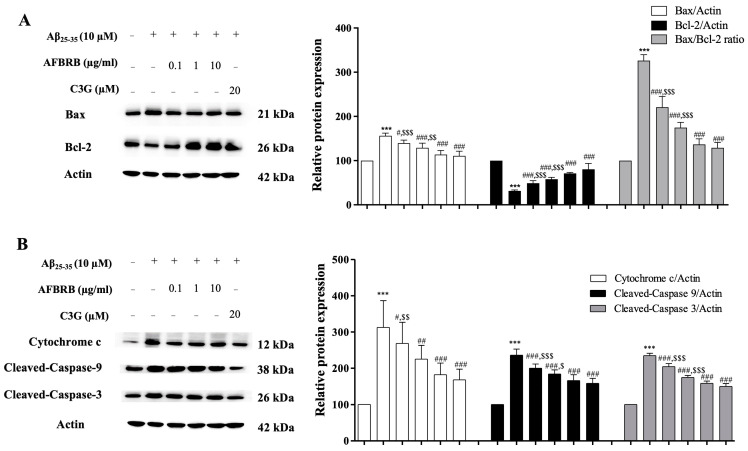
The protective effect of AFBRB and C3G on the Aβ_25-35_-induced mitochondrial death pathway in the SK-N-SH cells. The representative of AFBRB and C3G protected the upregulation of the Bcl-2, cytochrome c, cleaved caspase-9, and cleaved caspase-3, and the downregulation of Bax was determined using the Western blot analysis. The quantitative results of (**A**) Bax, Bcl-2, and Bax/Bcl-2 ratio and (**B**) cytochrome c, cleaved caspase-9, and cleaved caspase-3 proteins are displayed. Reprobing for β-actin served as the internal loading control. The data are expressed as mean ± SD of six independent experiments. *** *p* < 0.001 versus control group; ^#^ *p* < 0.05, ^##^ *p* < 0.01, and ^###^ *p* < 0.001 versus 10 µM Aβ_25–35_-treated group; ^$^ *p* < 0.05, ^$$^ *p* < 0.01, and ^$$$^ *p* < 0.001 versus C3G.

In order to examine the protective effect of AFBRB or C3G on the mitochondrial death pathway induced by Aβ_25–35,_ we first investigated the proteins related to the apoptosis pathway by Western blot analysis. Cells were pretreated with AFBRB or C3G for 2 h before the addition of 10 µM of Aβ_25–35_ for 24 h. Treatment with Aβ_25–35_ alone significantly increased the expression of Bax and decreased Bcl-2 expression (Figure 3A). Pretreatment with AFBRB at concentrations of 0.1, 1, or 10 µg/mL or 20 µM of C3G significantly decreased the levels of Bax expression and significantly increased Bcl-2 expression, and also significantly decreased the Bax/Bcl-2 ratio in a concentration-dependent manner. The Aβ_25–35_ treatment resulted in a significantly increased expression of cytochrome c, leading to the activation of cleaved caspase-9 and cleaved caspase-3 and subsequently increasing apoptosis. AFBRB or C3G significantly attenuated the expression of these proteins, as shown in Figure 3B. Our findings suggest that AFBRB can protect neuronal cells against Aβ_25–35_-induced apoptosis via the mitochondria-dependent caspase pathway. Interestingly, we found that AFBRB significantly alleviated cell death to a similar extent as C3G.

### 2.4. Effect of AFBRB on ER Stress in SK-N-SH Induced by Aβ_25–35_

**Figure 4:**
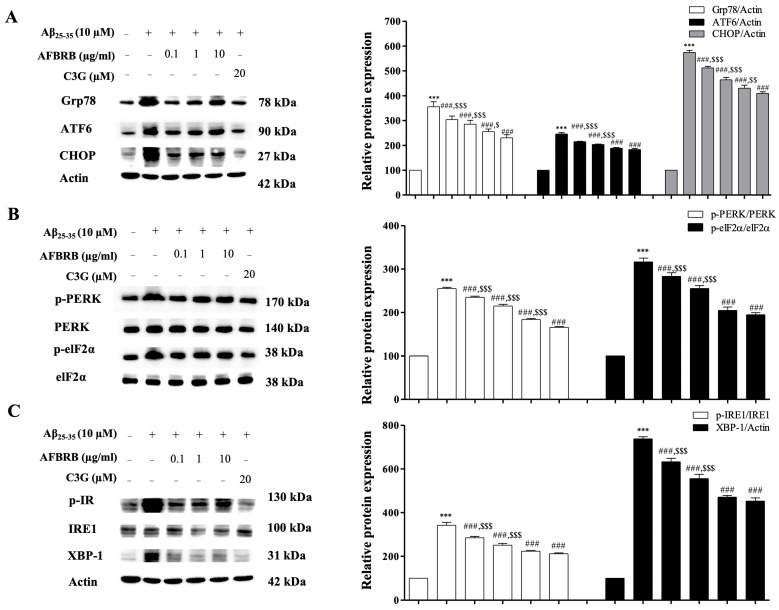
The protective effect of AFBRB and C3G on the Aβ_25–35_-induced ER stress in the SK-N-SH cells. The representative Western blot shows the expression and the quantitative analysis of (**A**) Grp78, CHOP, ATF6 (**B**) p-PERK/PERK, p-elF2α/ elF2α (**C**) p-IRE1/IRE1, and XBP-1. Reprobing for β-actin served as the internal loading control. The data are expressed as mean ± SD of six independent experiments. *** *p* < 0.001 versus control group; ^###^ *p* < 0.001 versus 10 µM Aβ_25–35_-treated group; ^$^ *p* < 0.05, ^$$^ *p* < 0.01, and ^$$$^ *p* < 0.001 versus C3G.

To confirm whether AFBRB attenuates the actions of Aβ_25–35_ with regard to the disturbance of ER function, the expression levels of Grp78, ATF6, CHOP, PERK, elF2α, IRE1α, and XBP-1 were evaluated by Western blot analysis. Cells were pretreated with AFBRB or C3G for 2 h, followed by incubation with Aβ_25–35_ for 24 h. The Aβ_25–35_ significantly increased the level of the ER stress marker Grp78 expression. Similar results were observed about the expression of ATF6, PERK, and IRE1, three ER transmembrane proteins involved in the ER stress pathways. Our data show that the expression levels of Grp78, cleaved ATF6, CHOP (Figure 4A), p-PERK, p-elF2α (Figure 4B), p-IRE1, and XBP-1 (Figure 4C) significantly increased in the Aβ_25-35_-treated cells when compared with the control group. However, pretreatment with AFBRB or C3G significantly downregulated the expression of these proteins. These results indicate that AFBRB attenuated the three major indicators of ER stress pathways.

**Figure 5:**
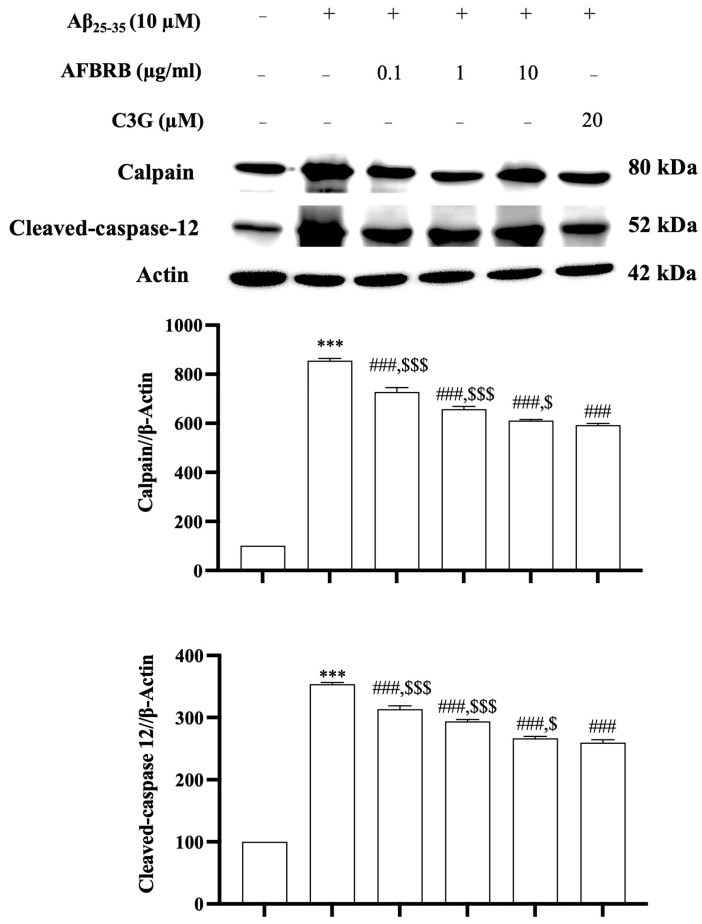
The protective effect of AFBRB and C3G on the Aβ_25–35_-induced apoptosis via ER stress in the SK-N-SH cells. The representative Western blot shows the expression and the quantitative analysis of calpain and cleaved caspase-12. Reprobing for β-actin served as the internal loading control. The data are expressed as mean ± SD of six independent experiments. *** *p* < 0.001 versus control group; ^###^ *p* < 0.001 versus 10 µM Aβ_25–35_-treated group; ^$^ *p* < 0.05, and ^$$$^ *p* < 0.001 versus C3G.

ER stress is related to the activation of calpain and caspase-12, resulting in apoptotic cell death. To determine the involvement of the apoptosis pathway in Aβ_25–35_-induced ER stress, the levels of calpain and cleaved caspase-12 expression were determined on SK-N-SH cells by Western blot analysis. Cells were pretreated with AFBRB at concentrations of 0.1, 1, or 10 µg/mL or 20 µM of C3G for 2 h before the addition of 10 µM of Aβ_25–35_ for 24 h. The results show that Aβ_25–35_ significantly increased the levels of calpain and cleaved caspase-12 expression, while pretreatment with AFBRB and C3G significantly decreased (*p* < 0.001) when compared to Aβ_25–35_ alone (Figure 5). These findings suggest that the effect of AFBRB and C3G may downregulate Aβ_25–35_-induced apoptosis through the ER stress pathway.

## 3. Discussion

In this study, we investigated the antioxidant effect of AFBRB on Aβ_25–35_-induced neurotoxicity in SK-N-SH cells. The major findings are as follows: (1) AFBRB improved oxidative status and reduced ER stress in SK-N-SH cells, and (2) AFBRB ameliorated apoptosis in SK-N-SH cells.

AD is a progressive neurodegenerative disorder in the aging population and is mainly characterized by the pathological hallmarks of extracellular accumulation of beta-amyloid (Aβ) plaques and intraneuronal tau-containing neurofibrillary tangles in the brain [3,27]. The accumulation of Aβ also plays a critical role in accelerating the progression of AD by promoting mitochondrial dysfunction, oxidative stress, inflammatory response, and neuronal death [28]. Several studies have reported that Aβ_25–35_, the fragment located in position 25–35, represents the biologically active region of Aβ that contains large β-sheet aggregated structures and retains the toxicity of the full-length peptide [29,30,31,32]. Several studies have also highlighted that Aβ_25–35_ is highly toxic to neurons. Aβ_25–35_ can induce neurotoxicity leading to neuronal cell death through many mechanisms, including the generation of neuronal oxidative stress, and the induction of apoptosis mediated by oxidative stress and the ER stress pathway [11,33,34,35].

The anthocyanin-rich fraction of black rice bran extract contains high anthocyanin content that prevents and inhibits the development of several chronic diseases via anti-apoptosis, anti-ER stress mechanisms, and antioxidants. C3G has been identified as having remarkable properties as a strong antioxidant agent, having anti-inflammatory and anti-neurodegenerative effects [33,36,37]. The findings of this study demonstrate that the AFBRB could protect SK-N-SH cells against oxidative stress, ER Stress, and the actions of the neuronal apoptosis pathway. Our results from the MTT assay found that the AFBRB could improve the number of viable cells in a concentration-dependent manner. Excessive amounts of Aβ can generate the production of ROS, leading to oxidative stress, and play a key role in Aβ-induced neuronal cell death [11,34,38]. In agreement with these findings, this study demonstrated that Aβ_25–35_ significantly induced the generation of ROS; however, pretreatment of SK-N-SH cells with the AFBRB attenuated the changes mentioned earlier, suggesting that the neuroprotective action of AFBRB may be related to its antioxidant ability. It was also found that excessive levels of ROS lead to mitochondrial dysfunction, lipid peroxidation, and apoptosis [34,39]. To evaluate the molecular mechanisms associated with the Aβ_25–35_-induced mitochondrial death pathway changes, we investigated the effect of AFBRB on Aβ_25–35_-induced levels of expression of the proteins Bax, Bcl-2, cytochrome c, cleaved caspase-3, and cleaved caspase-9 protein levels using Western blotting techniques. The actions of the Bcl-2 family of proteins (Bax is one of the pro-apoptotic proteins that regulate programmed cell death, whereas the Bcl-2 is an anti-apoptotic protein) stabilize mitochondrial membrane permeability and inhibit the release of cytochrome c into the cytosol [40]. The balance between Bax and Bcl-2 proteins is important, resulting in either cell survival or death. During the initial phase of apoptosis, oxidative stress can activate the imbalance of Bcl-2/Bax in the mitochondrial membrane, which causes the loss of mitochondrial membrane potential and the release of cytochrome c into the cytosol [41,42]. We found that Aβ_25–35_ significantly increased the expression of Bax, decreased the expression of Bcl-2, and increased the Bax/Bcl-2 ratio. These findings corresponded with those of previous studies in that these proteins play an important role in mitochondrial apoptosis caused by oxidative stress. Pretreating cells with AFBRB resulted in a decreased expression of Bax and an increased expression of Bcl-2; these potentially adaptive responses include downregulation of the Bax/Bcl-2 ratio. Normally, cytochrome c is located in the intermembrane space of the mitochondrion. The release of cytochrome c from the mitochondrion into the cytosol activates the caspase-9 and caspase-3 and, subsequently, the intrinsic apoptosis pathway [43,44]. Our study found that Aβ_25–35_ significantly increased the level of cytochrome c and subsequently increased the expression of cleaved caspase-9 and cleaved caspase-3. Pretreatment with AFBRB reversed these Aβ_25–35_-induced changes in the apoptosis pathway. Interestingly, we found that AFBRB significantly alleviated cell death in SK-N-SH cells in cases of toxicity induced by Aβ_25–35_ to a similar extent as C3G treatment. In addition, previous studies have proved that Aβ_25–35_ stimulates the production of ROS, resulting in oxidative stress, impairment of cell function, and activation of neuronal death via mitochondrial apoptosis and the ER stress pathway [11,45]. The ER plays a crucial role in protein folding and modification. Many conditions can disturb the functioning of the ER; for example, the depletion of calcium, changes in glycosylation, oxidative stress via initiation of the unfolding of proteins in the ER lumen, disruption of the ER homeostasis, and activation of a complex signaling network, which initiates the unfolded protein response (UPR), leads to ER stress. The UPR is initiated by the three transmembrane stress sensors, specifically ATF6, PERK, and IRE1, which are activated by Grp78, an ER-resident chaperone known as an ER stress marker. The binding of unfolded proteins with Grp78 results in its detachment from the three stress sensors, followed by the translocation and transcription of ATF6 into the nucleus. PERK phosphorylates elF2α in order to activate protein phosphorylation and translation. The activation of IRE1 leads to XBP-1 splicing, transcriptional activation of chaperones, and stimulates the degradation of proteins. These three stress sensors can regulate CHOP, a pro-apoptotic protein intrinsic to the caspase cascade pathway. Recently, it has been considered that ER stress is potentially involved in the development of AD [46,47]. Previous studies have reported that Aβ_25–35_ overload can induce ER stress, resulting in neuronal apoptosis. Moreover, excess levels of ROS can disturb intracellular calcium homeostasis, which may be involved in ER function and induce ER stress [48,49,50]. Therefore, the protective effects of AFBRB against Aβ_25–35_-induced ER stress were investigated. The results indicate that pretreatment with AFBRB significantly decreased the levels of expression of Grp78, cleaved ATF6, CHOP, p-PERK, p-elF2α, p-IRE1, and XBP-1. Moreover, calpain, an intracellular calcium-dependent cysteine protease, is activated by elevated intracellular concentrations of calcium [51,52]. Calcium can activate calpain and induce the cleavage of caspase-12. Caspase-12 is the ER’s resident protease protein that activates the caspase-9 protein, subsequently leading to the activation of caspase-3, a process associated with the mitochondrial death pathway [53,54]. Thus, the findings of this study confirm that Aβ_25–35_ significantly increased the levels of calpain and cleaved caspase-12 in SK-N-SH cells, leading to ER stress-associated apoptosis. Therefore, AFBRB mediated the inhibition of calpain and cleaved caspase-12 expression, suggesting that the therapeutic implementation of the protective effects of AFBRB may be a critical strategy in the efforts to prevent ER stress-induced Aβ_25–35_. Moreover, various studies showed that the rice varieties such as Kum Chao CMU 107 (CMU 107), Sang 5 CMU (SNG 5), Pieisu 1 CMU (PES 1), Kum Akha 1 CMU (KAK 1), Bien Koo 5 CMU (BKU 5), K2 CMU (K2), K4 CMU (K4), Kum Doi Saket (KDK) [55], and purple glutinous rice (*O. sativa* L. *indica*) [11] have numerous benefits by their antioxidant activities and anti-inflammatory properties. Our study demonstrated that AFBRB is the new potential agent in attenuating Aβ_25–35_-induced oxidative stress, ER stress, and apoptosis in SK-N-SH cells. AFBRB might be developed into a new functional food in the future, but it is necessary to prove in animal models and clinical trials in future studies.

## 4. Materials and Methods

Shrimp oil might be the new alternative treatment that can enhance the efficiency of standard treatment.

### 4.1. Cell Culture

Cells from a human neuroblastoma cell line (SK-N-SH) were grown in minimum essential medium (MEM) supplemented with 10% heat-inactivated FBS and 1% penicillin–streptomycin (GIBCO, Thermo Fisher Scientific, Waltham, MA, USA) and maintained at 37 °C in 95% humidified air, 5% CO_2_ in an incubator. The medium was changed every 2 days and the cells reached approximately 80% confluence.

### 4.2. Chemicals

Aβ_25-35_, cyanidin-3-*O*-glucoside (C3G), 3-(4,5-dimethylthiazol-2-y1)-2,5-diphenyltetrazolium bromide (MTT), and 2′,7′-dichlorofluorescin diacetate (H_2_DCFDA) were purchased from Sigma (St. Louis, MO, USA). The antibodies used for the Western blot analysis were as follows: anti-Bax, anti-Bcl-2, anti-cytochrome c, anti-β-actin, anti-mouse IgG peroxidase-conjugated secondary antibody, and anti-rabbit IgG peroxidase-conjugated secondary antibody (Millipore, Bedford, MA, USA), anti-cleaved caspase-3 and anti-cleaved caspase-9, anti-Grp78, anti-CHOP, anti-PERK, anti-p-PERK, anti-eIF2α, anti-p-eIF2α, anti-IRE1α, anti-p-IRE1α, anti-calpain (Cell Signaling Technology, Danvers, MA, USA), anti-ATF6, anti-XBP-1 (Santa Cruz Biotechnology, Dallas, TX, USA), and anti-cleaved caspase-12 (Abcam, Cambridge, UK).

### 4.3. Preparation of Aggregated Aβ_25–35_

Aβ_25–35_ peptide powder was reconstituted in sterile water at a concentration of 1 mM and stored at −80 °C. Before use in any protocol, Aβ_25–35_ was subsequently diluted to a concentration of 10 µM and maintained at 37 °C for 5 days to enable the formation of aggregated Aβ.

### 4.4. Preparation of Anthocyanin-Rich Fraction of Black Rice Bran (AFBRB)

The Luem Pua rice was cultivated in the experimental plant cultivation area of Khao Kho, located in Phetchabun Province (latitude 16.370592, longitude 101.132324) in lower northern Thailand. The rice is cataloged with the accession number IRGC 48315, available in the International Rice Research Institute (IRRI) Gene Bank. The rice bran samples were extracted using a 4:1 ratio of ethanol–water solution at room temperature to yield ethanol extract. The extract was filtered and evaporated under reduced pressure at 40 °C to obtain the residual aqueous fraction. This fraction was then defatted by partitioning with hexane three times to obtain the dried defatted extract. The extract was fractionated by column chromatography, having Amberlite XAD7HP as a stationary phase to obtain an anthocyanin-rich fraction. Anthocyanin contents in the fraction were determined using high-pressure liquid chromatography (HPLC) with a Thermo C18 column (4.6 × 150 mm, 5 µm) and rhodamine B as an internal standard. One gram of the black rice bran extract was found to contain 585 mg of cyanidin-3-*O*-glucoside and 24 mg of peonidin-3-*O*-glucoside (Supplementary Materials).

### 4.5. Measurement of Cell Viability

The cell viability was evaluated using MTT reduction assay by measuring mitochondrial function [56]. SK-N-SH cells were seeded at a density of 2 × 10^5^ cells/mL in 96-well plates and incubated at 37 °C, 5% CO_2_, and 95% air humidified for 24 h. Cells were pretreated with 0.1, 1, or 10 µg/mL of AFBRB or 20 µM of C3G for 2 h before the addition of 10 µM of Aβ_25–35_. After incubation for 24 h, 50 µL of MTT solution (1 mg/mL) was added to each well and incubated further at 37 °C for 2 h. Thereafter, the medium was discarded. Dimethyl sulfoxide (DMSO) 100 µL was added to each well to dissolve the formazan crystals, and the absorbance was measured at 570 nm using a microplate reader (BioTek Instruments, Inc., Winooski, VT, USA). The percentage of cell viability was calculated as follows: A570 nm of treated cells/A570 of control cells × 100.

### 4.6. Measurement of Reactive Oxygen Species (ROS)

The production of intracellular ROS was evaluated using 2′,7′-dichlorofluorescin diacetate (DCFDA assay) [56]. SK-N-SH cells were cultured in a 96-well plate at a density of 2 × 10^5^ cells/mL at 37 °C overnight in the dark. Cells were pretreated with 0.1, 1, or 10 µg/mL of AFBRB or 20 µM of C3G for 2 h before the addition of 10 µM of Aβ_25–35_ for 24 h. Then, the medium was discarded and (1×) PBS containing 20 µM of H_2_DCF-DA was added to each well. The cells were then incubated at 37 °C for 2 h. The fluorescent absorbance was measured at an excitation wavelength of 485 nm and an emission wavelength of 535 nm using a fluorescent microplate reader (DTX800, Beckman Coulter, Vienna, Austria).

### 4.7. Western Blot Analysis

For analysis of the expression of the proteins, SK-N-SH cells were seeded at a density of 5 × 10^5^ cells/mL in a 60 mm culture dish at 37 °C, 5% CO_2_, 95% air humidified, and left overnight. Cells were pretreated with 0.1, 1, or 10 µg/mL of AFBRB or 20 µM of C3G for 2 h before the addition of 10 µM of Aβ_25–35_ for 24 h. Subsequently, the cells were collected and lyzed using a lysis buffer (1% NP-40, 1% sodium deoxycholate, 0.1% sodium dodecyl sulfate, 40 mM β-glycerophosphate, 50 mM sodium fluoride, 2 mM sodium orthovanadate, and a cocktail of protease inhibitors), followed by centrifugation at 4 ºC, 13,000 rpm for 20 min. The supernatant was collected and the total protein concentration was determined using the Bradford protein assay (BioRad, Hercules, CA, USA). Protein samples (25 µg) were electrophoresed in a 10%-15% SDS polyacrylamide gel and transferred to the polyvinylidene fluoride (PVDF) membrane (Immobilon-P, Millipore, Bedford, MA, USA). The membranes were blocked with blocking buffer (0.1% tween-20 in Tris-buffered saline, pH 7.4, containing 5% skim milk) for 2 h at room temperature and probed with primary antibodies (anti-Bax (1:1000; Cat No. #AF0120, Affinity Biosciences, Cincinnati, OH, USA), anti-Bcl-2 (1:1000; Cat No. #AF6139, Affinity Biosciences), anti-cytochrome c (1:1000; Cat No. MAB1800, Merck Millipore), anti-cleaved caspase-3 (1:1000; Cat No. 9661T, Cell Signaling Technology), anti-cleaved caspase-9 (1:1000; Cat No. 9507S, Cell Signaling Technology), anti-Grp78 (1:1000; Cat No. sc-13539, Santa Cruz Biotechnology), anti-CHOP (1:1000; Cat No. 2895S, Cell Signaling Technology), anti-ATF6 (1:1000; Cat No. 09-069, Merck Millipore), anti-PERK (1:1000; Cat No. 3192S, Cell Signaling Technology), anti-p-PERK (1:1000; Cat No. 3179S, Cell Signaling Technology), anti-eIF2α (1:1000; Cat No. 2103S, Cell Signaling Technology), anti-p-eIF2α (1:1000; Cat No. 9721S, Cell Signaling Technology), anti-IRE1α (1:1000; Cat No. 3294S, Cell Signaling Technology), anti-p-IRE1α, (1:1000; Cat No. ab48187, Abcam), anti-XBP-1 (1:1000; Cat No. sc-32136, Santa Cruz Biotechnology), anti-calpain (1:1000; Cat No. 2556, Cell Signaling Technology), and anti-caspase-12 (1:1000; Cat No. ab18766, Abcam) at 4 °C overnight, followed by incubation with mouse or rabbit IgG peroxidase-conjugated secondary antibodies. Finally, the membranes were incubated with ECL substrate solution to visualize the chemiluminescent bands. The protein bands were detected by the Omega Lum™ W Imaging System 81-12120-00 (Aplegen Gel Company, Inc., San Francisco, CA, USA), and the densitometry analysis was normalized using β-actin using Image-J^®^ software version 1.53.

### 4.8. Statistical Analysis

All values are presented as mean ± SD for the six independent experiments. Data were analyzed by one-way analysis of variance (ANOVA) followed by post hoc Tukey’s multiple tests to investigate comparisons between experimental groups by using Graphpad Prism 8.0 software (Graphpad, San Diego, CA, USA). A value of *p* < 0.05 was considered statistically significant.

## 5. Conclusions

In conclusion, our data indicated that the anthocyanin-rich fraction of black rice bran extracted by ethanol and water attenuated Aβ_25–35_-induced oxidative stress, neurotoxicity, ER stress, and apoptosis in SK-N-SH cells to a similar extent to treatment with C3G. This improvement was mediated through antioxidant activity. The proposed mechanisms involve the reduction in ROS in cells and the subsequent inhibition of the release of cytochrome c, followed by downregulation of the caspase-9 and caspase-3 proteins, leading to the prevention of the mitochondrial death pathway. This process occurs together with the inhibition of three stress sensors of ER stress, downregulation of the Grp78, CHOP proteins, and reduction in calpain and caspase-12 proteins, leading to the attenuation of apoptosis via the ER stress pathway. This study provides evidence that AFBRB may be considered as an alternative therapeutic agent for the prevention of the progression of neurodegenerative diseases.

## Data Availability

The original contributions presented in this study are included in the article/Supplementary Materials. Further inquiries can be directed to the corresponding author.

## Supplementary Materials

The following supporting information can be downloaded at: https://www.mdpi.com/article/10.3390/ph17081039/s1, Figure S1. HPLC chromatograms of (A) the cyanidin 3-*O*-glucoside standard and (B) the anthocyanin-rich fraction of black rice bran (AFBRB) of the rice variety Luem Pua. The AFBRB contains two main components, using a photodiode array as a detector at 254 nm. Therefore, sugars, expected to be the main non-phenolic polar compounds and likely to co-elute in the Amberlite fractionation, did not appear in this chromatogram; Figure S2. Mass spectra obtained from positive mode electrospray ionization of chemical constituents in AFBRB (A) peak 1 and (B) peak 2, corresponding to cyanidin-3-*O*-glucoside and peonidin-3-*O*-glucoside, respectively.
