# Supplementary material for: Anthocyanin-Rich Fraction of Black Rice Bran Extract Protects against Amyloid β-Induced Oxidative Stress, Endoplasmic Reticulum Stress, and Neuronal Apoptosis in SK-N-SH Cells
Source: Pharmaceuticals (Basel). 2024 Aug 7;17(8):1039. doi: 10.3390/ph17081039 (PMC11357448; doi:10.3390/ph17081039)
Supplement: Supplementary file 1 [file pharmaceuticals-17-01039-s001.zip › pharmaceuticals-3115049-supplementary.pdf]

Supporting Information

Anthocyanin-Rich Fraction of Black Rice Bran Extract Protects Against Amyloid β-Induced Oxidative Stress, Endoplasmic Reticulum Stress, and Neuronal Apoptosis in SK-N-SH Cells

Sivanan Sivasinprasasn[1,2], Jiraporn Tocharus[3], Sugunya Mahatheeranont[4,5], Sarun Nakrat[4,5], Chainarong Tocharus[1,*]

[1] Department of Anatomy, Faculty of Medicine, Chiang Mai University, Chiang Mai 50200, Thailand; dymesiva@gmail.com (S.S.)
[2] Office of Research Administration, Chiang Mai University, Chiang Mai 50200, Thailand
[3] Department of Physiology, Faculty of Medicine, Chiang Mai University, Chiang Mai 50200, Thailand; jtocharus@gmail.com (J.T.)
[4] Department of Chemistry, Faculty of Science, Chiang Mai University, Chiang Mai, Thailand; sugunya.w@gmail.com (S.M.); sarun_n@cmu.ac.th (S.N.)
[5] Center of Excellence for Innovation in Chemistry, Faculty of Science, Chiang Mai University, Chiang Mai, Thailand

*Correspondence: chainarong.t@cmu.ac.th (C.T.)

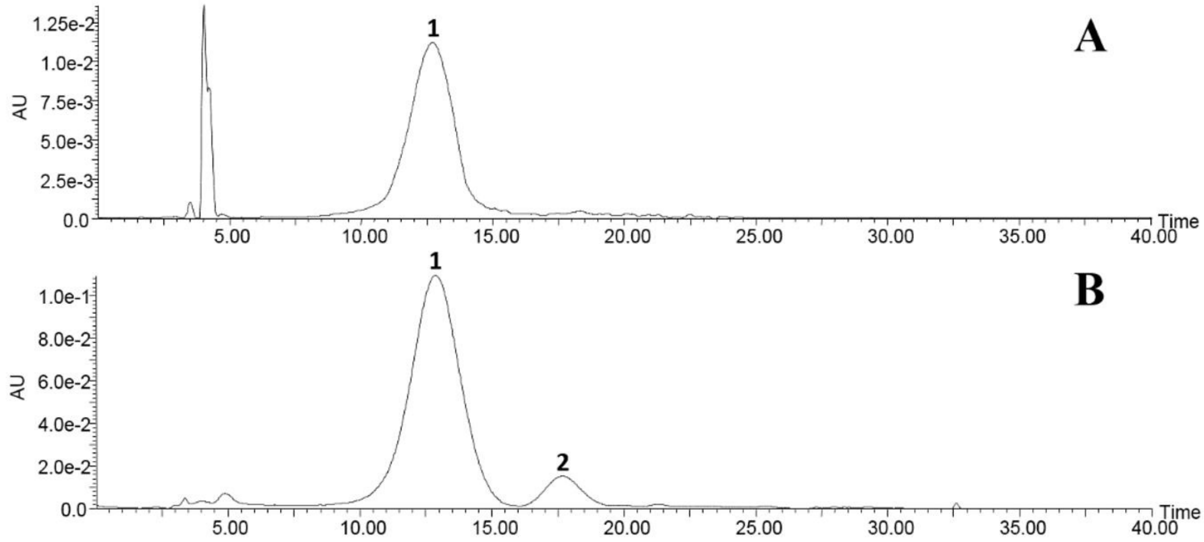

Figure S1. HPLC chromatograms of (**A**) the cyanidin 3-*O*-glucoside standard and (**B**) the anthocyanin-rich fraction of black rice bran (AFBRB) of the rice variety Luem Pua. The AFBRB contains two main components, using a photodiode array as a detector at 254 nm. Therefore, sugars, expected to be the main non-phenolic polar compounds and likely to co-elute in the Amberlite fractionation, did not appear in this chromatogram.

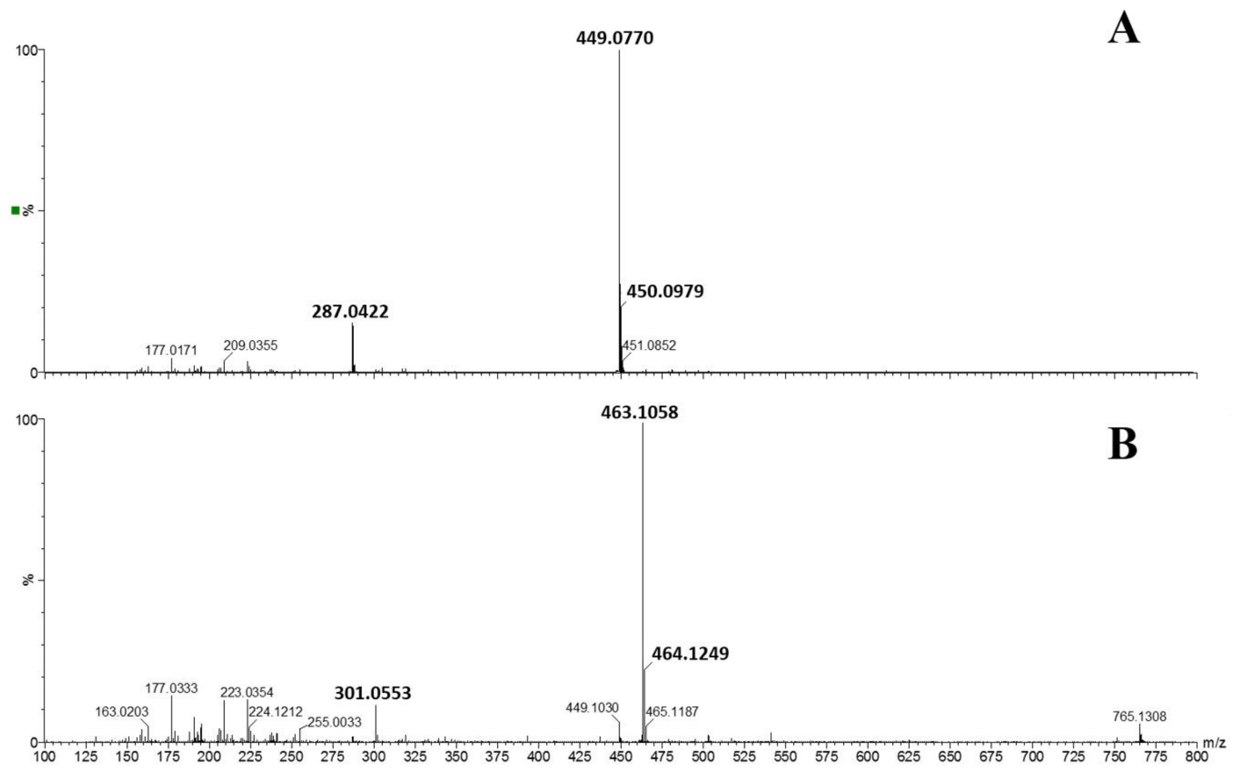

Figure S2. Mass spectra obtained from positive mode electrospray ionization of chemical constituents in AFBRB (**A**) peak 1 and (**B**) peak 2, corresponding to cyanidin-3-*O*-glucoside and peonidin-3-*O*-glucoside, respectively.
